# Assessment of molecular and morphological dynamics during long-time *in vitro* cultivation of cryopreserved human ovarian tissue: risk of genetic alterations

**DOI:** 10.3389/fendo.2024.1463614

**Published:** 2025-05-02

**Authors:** Wanxue Wang, Plamen Todorov, Evgenia Isachenko, Gohar Rahimi, Markus Merzenich, Nina Mallmann-Gottschalk, Yang Zhou, Jilong Yao, Xuemei Li, Volodimir Isachenko

**Affiliations:** ^1^ Department of Obstetrics and Gynaecology, Medical Faculty, Cologne University, Cologne, Germany; ^2^ Reproductive Medicine Centre, Shenzhen Maternity and Child Healthcare Hospital, Shenzhen, China; ^3^ Department of Reproductive Biotechnology and Cryobiology of Gametes Institute of Biology and Immunology of Reproduction of Bulgarian Academy of Sciences (BAS), Sofia, Bulgaria; ^4^ Medizinisches Versorgungszentrum AMEDES für IVF- und Pränatalmedizin in Köln GmbH, Cologne, Germany; ^5^ Department of Gynecology, Zhongshan Hospital, Fudan University, Shanghai, China

**Keywords:** human ovarian tissue cryopreservation, *in vitro* culture, Single Nucleotide Polymorphism (SNP), Insertions-Deletions (InDel), protein kinase inhibitor gamma (PKIG), SE (skipped exon), negative effect on genes expression

## Abstract

**Background:**

Cryopreservation of human ovarian tissue is a technology for patients undergoing aggressive anticancer treatments. This technology includes the following stages: saturation by permeable cryoprotectants, freezing, thawing, removal of cryoprotectants, as well as tissues *in vitro* or *in situ* culture.

**Objective:**

Evaluation of quality of tissue after cryopreservation and *in vitro* culture with the aim of detection of genetic and molecular changes in cells.

**Methods:**

Ovarian tissue was frozen in 6% ethylene glycol and 6% dimethyl sulfoxide with speed of cooling 0.3°C/min and thawed at 100°C. After removal of cryoprotectants tissue fragments were *in vitro* cultured with the soluble extract of basement membrane protein (Matrigel) 3-D culture system for 7 days. Morphological and functional assessments were conducted using microscopic observation and RNA-Sequencing. Comparative analysis of tissue morphology before and after culture was performed with bioinformatics for gene expression and variant analysis, including functional annotation and study of protein-protein interaction.

**Results:**

DNA and RNA analyses after cultivation indicated a rise in gene fusion and alternative splicing events, potentially affecting gene expression and cellular functions.

**Conclusion:**

Long-time *in vitro* culture of human ovarian tissue results in substantial changes in its morphology and genetic alteration.

## 1 Introduction

Ovarian tissue cryopreservation can help women who are undergoing cancer treatment, autoimmune diseases, or other treatments with radiation or/and aggressive chemotherapy, to preserve their ovarian tissue for function in future (1, 2). The key of this technology is a surgical extraction of ovarian tissue using laparoscopy, freezing and storage them in liquid nitrogen (3, 4). The main purpose of cryopreservation of ovarian tissue before anti-cancer treatment is preservation of primordial follicles, which after anti-cancer treatment and thawing can be used for auto-transplantation (5).

In fact, anti-cancer therapy may damage the ovarian tissue and lead to decreased or complete loss of fertility (6, 7). In the same time, cryopreservation of this tissue and auto-transplantation can increase the chances of patients to be pregnant (8). However, the success of the ovarian tissue auto-transplantation is depended from various factors: quantity and quality of follicles in this tissue, quality of tissue freezing and thawing as well as an age of patient (9, 10). In addition, this technology may also be limited by financial and ethical considerations for patients (11, 12).

*In vitro* culture of ovarian tissue is a technique of cultivating of this tissue under specific culture conditions (13). This technology can be used to study issues related to ovarian development, reproductive physiology, and reproductive toxicology. Cryopreservation includes the following stages: saturation by permeable cryoprotectants, freezing, thawing, removal of cryoprotectants, and *in vitro* or *in situ* culture. The last stage of cryopreservation (*in vitro* culture) is aimed to evaluate a quality of whole process of cryopreservation (14, 15). Once the ovarian tissues are obtained, it should be immediately placed in a nutrient solution to maintain the cells viability. Methods of handling of ovarian tissue for *in vitro* culture include removing of surrounding tissues (medulla) and blood vessels as well as dividing of cells into slices or pieces. The processed tissues should be placed in a cell culture medium. Special culture conditions, such as appropriate oxygen levels, hormone concentrations in the nutrient solution, and pH values, can be achieved by adjusting of gas and composition of the culture medium in incubators for cell culture (16).

Recent years can be characterized by increasing of attention to the molecular mechanisms of ovarian tissue *in vitro* culture. It was found that hormone concentrations at *in vitro* culture has a significant impact on the growth and development of ovarian tissue. Growth factors and cytokines can promote proliferation and differentiation of ovarian tissue. Extracellular matrix is a complex structure outside of cells, playing a crucial role in cell growth and differentiation (17).

It can be summarized that the molecular mechanisms of ovarian tissue *in vitro* culture are very complex, requiring a comprehensive consideration of various factors, including hormones, growth factors, extracellular matrix, and cellular metabolic processes such as autophagy and apoptosis (18).

The aim of our investigations was the evaluation of genetic risks and molecular alterations in human ovarian tissue during *in vitro* culture.

## 2 Methods

**Figure 1:**
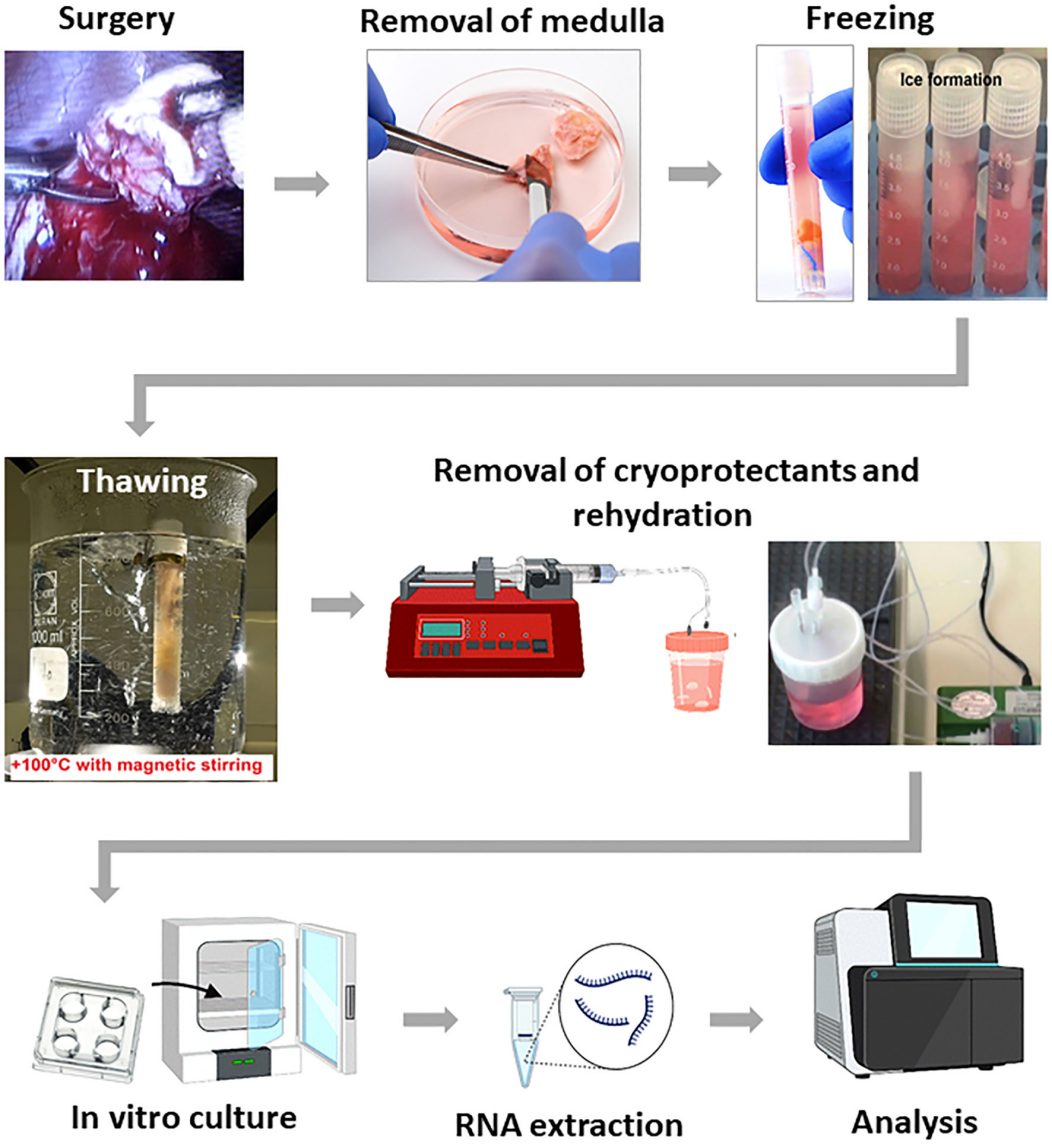
Schema of ovarian tissue cryopreservation, *in vitro* culture and analysis of viability.

Except where otherwise stated, all chemicals were obtained from Sigma (Sigma Chemical Co., St. Louis, MO, USA). The primary experimental procedure of our experiments is shoved in **Figure 1**.

### 2.1 Ovarian tissue collection, freezing and thawing

The study adhered to the stipulations of the Helsinki Declaration and received approval from the Ethical Review Committee of the University of Cologne (License numbers 999,184 and 13-147) and by the Bulgarian Ethics Committee. The cryopreservation facility at the Cologne University Maternal Hospital was used to store all ovarian tissues collected during the sampling phase. Moreover, the Ethics Committee approved a protocol that allows the use of 10% of ovarian tissues collected from patients for research. Informed consent was obtained from 6 subjects of age 23 to 41 years (median age 36.2 years) involved in the study.

Fresh ovarian tissue samples by 32-34°C were transported to laboratory in Leibovitz-15 culture media (Irvine Sci., Santa Ana, CA, USA) enriched with 5% Serum Substitute Supplement (SSS, Irvine Sci.). In the laboratory, ovarian medulla was partially separated from cortex using forceps and a no. 22 scalpel (19–21).

Cryopreservation of ovarian tissue was performed according to our previously published protocol (19–21). On the day of freezing, pieces of ovarian tissue were placed at room temperature in 20 mL freezing medium composed of basal medium supplemented with 6% dimethyl sulfoxide, 6% ethylene glycol, and 0.15 M sucrose. Then, pieces were put into standard 5 mL cryo-vials (Thermo Fisher Scientific, Rochester, NY, USA), which were previously filled by freezing medium and frozen in IceCube 14S freezer (SyLab, Neupurkersdorf, Austria). The cryopreservation program included the following stages: (1) starting temperature was −6°C to -8°C; (2) samples were cooled from −6°C to −34°C at a rate of 0.3 °C/min; and (3) at −34 °C cryo-vials were plunged into liquid nitrogen. The freezing protocol for cryopreservation of this ovarian tissue included an auto-seeding step at -6°C to -8°C.

Thawing of tissue (19–21) was achieved by holding the vial for 30s at room temperature, followed by immersion in a 100°C (boiling) water for 60 s, and expelling the contents of the vial into the solution for removal of cryoprotectants. The exposure time in the boiling water was visually controlled by the presence of ice in the medium; as soon as the ice reached 2 to 1 mm apex, the vial was removed from the boiling water, at which point the final temperature of the medium was between 4°C and 10°C. Within 5 to 10s after thawing, the tissue from cryo-vials were expelled into 10 mL thawing solution (basal medium containing 0.5 M sucrose) in a 100 mL specimen container (Sarstedt, Nuembrecht, Germany). After thawing tissue fragments were used for following analysis.

### 2.2 Experimental design and *in vitro* culture

Pieces of Group 1 (control, n = 18) were used for analysis just after cryopreservation. Pieces of Group 2 (experimental group, n = 18) were placed for *in vitro* culture. This *in vitro* culture was performed in accordance with the method previously described by Higuchi et al. (22) with minor changes.

Ovarian tissue *in vitro* culture was performed in 1.0 ml of culture medium at 37°C, 5%CO_2_ in air in 4-well Peri dishes. This medium included the *in vitro* growth (IVG) medium consisting of αMEM (Invitrogen/Termo Fisher Sci., Schwerte, Germany) and supplemented with 5% FBS, 30 ng/ml Activin A (Sigma-Aldrich/Merck KGaA, Darmstadt, Germany), 100 mIU/ml rhFSH (Merck KGaA, Darmstadt, Germany), 5 μg/ml insulin, 5 μg/ml transferrin, 5 ng/ml selenium (Sigma-Aldrich). Half of the culture media was replaced every day.

Pieces of Group 2 (experimental group, n=18) were placed onto a floating membrane filter (0.4 μm HTTP; Merck Millipore/Merck KGaA, Darmstadt, Germany) (1 piece/membrane). Then 3-D culture system was formed. For this aim, soluble extract of basement membrane protein (23), Corning Matrigel Matrix (Life Sci., San Diego, CA, USA) was diluted with IVG medium (1:1) and 5 μl were placed on a floating membrane filter to make a small drop (1 drop/membrane). Then, ovarian pieces were placed on top of each drop (1 piece/drop), cultured for 7 days. Half of the culture media was replaced every day.

### 2.3 Morphology of tissues

To delve into the tissue (Group 1, n=12 and Group 2, n=12) microanatomy and morphological attributes, it was employed the time-tested paraffin sectioning technique for microscopic observation. Tissue samples were initially submerged in a 10% neutral-buffered formalin solution for 12-24 hours to prevent autolysis and structural degradation. After fixation, tissues were progressively immersed in graded concentrations of alcohol solutions to remove inherent water content. Dehydrated tissues were then placed in clearing agent chloroform to achieve transparency. For impregnation tissue samples were subsequently submerged in molten paraffin, ensuring thorough paraffin penetration. Paraffin-impregnated tissues were cast into molds and cooled at room temperature, forming paraffin blocks. Using a rotary microtome, thin sections (4 microns in thickness) were sliced from the paraffin blocks. Then sections were placed on glass slides and subjected to staining technique by Hematoxylin and Eosin (HE staining). After staining, sections were observed under a microscope, capturing clear images to reveal cellular structures and morphological features.

### 2.4 RNA-Sequencing

A total of 12 RNA samples (Group 1, n=6 and Group 2, n=6), were used for deep sequencing analysis. These samples were prepared for library construction using the DNA Nanoball (DNB) Prep Kit and were subsequently sequenced in paired-end mode on the DNBSEQ technology platform. The raw data obtained, saved in fq.gz format, has been uploaded to the Sequence Read Archive database at the National Center for Biotechnology Information (specific links can be found in the Data Availability section). In the data processing workflow, it was first conducted quality control steps to ensure the removal of low-quality and potentially contaminating sequences. The specific quality control procedures are as follows: (1) Removal of adapter sequences possibly introduced during library preparation; (2) Quality assessment of raw reads using FastQC, excluding reads with an N content of more than 10%; (3) Quality trimming and ensuring the removal of bases with a Q score (quality value) greater than 50%. Subsequently, the clean data was used for subsequent transcriptome assembly and differential expression analysis.

### 2.5 Bioinformatics analysis

In this study, it was employed a series of bioinformatics techniques to analyze the raw RNA-seq data. Initially, it was received the raw sequencing data in FASTQ format and performed preliminary processing through customized Perl scripts to ensure high data quality. To estimate gene expression levels, it was adopted the FPKM method, which accounts for the effects of sequencing depth and gene size on fragment counts. For further variant analysis, it was deeply analyzed the Single Nucleotide Polymorphism (SNP) data using tools Genome Analysis Toolkit (GATK) and programs for interacting with high-throughput sequencing data (Samtools). Additionally, it was used KEGG (Kyoto Encyclopedia of Genes and Genomes) Orthology-Based Annotation System (KOBAS) for enrichment analysis of variant sites. The online tool Chiplot was also used for statistical visualization and supplementary analysis of alternative splicing. Subsequently, it was conducted functional annotations using tools Gene Set Enrichment Analysis (GSEA), Gene Ontology (GO), KEGG, delving deeply into the functions of genes and proteins. For further exploration of interactions between proteins, it was carried out protein-protein interaction (PPI) analysis using Cytoscape and the STRING database. For alternative splicing analysis, it was utilized tools SpliceSeq and rMATS (24).

## 3 Results

**Figure 2:**
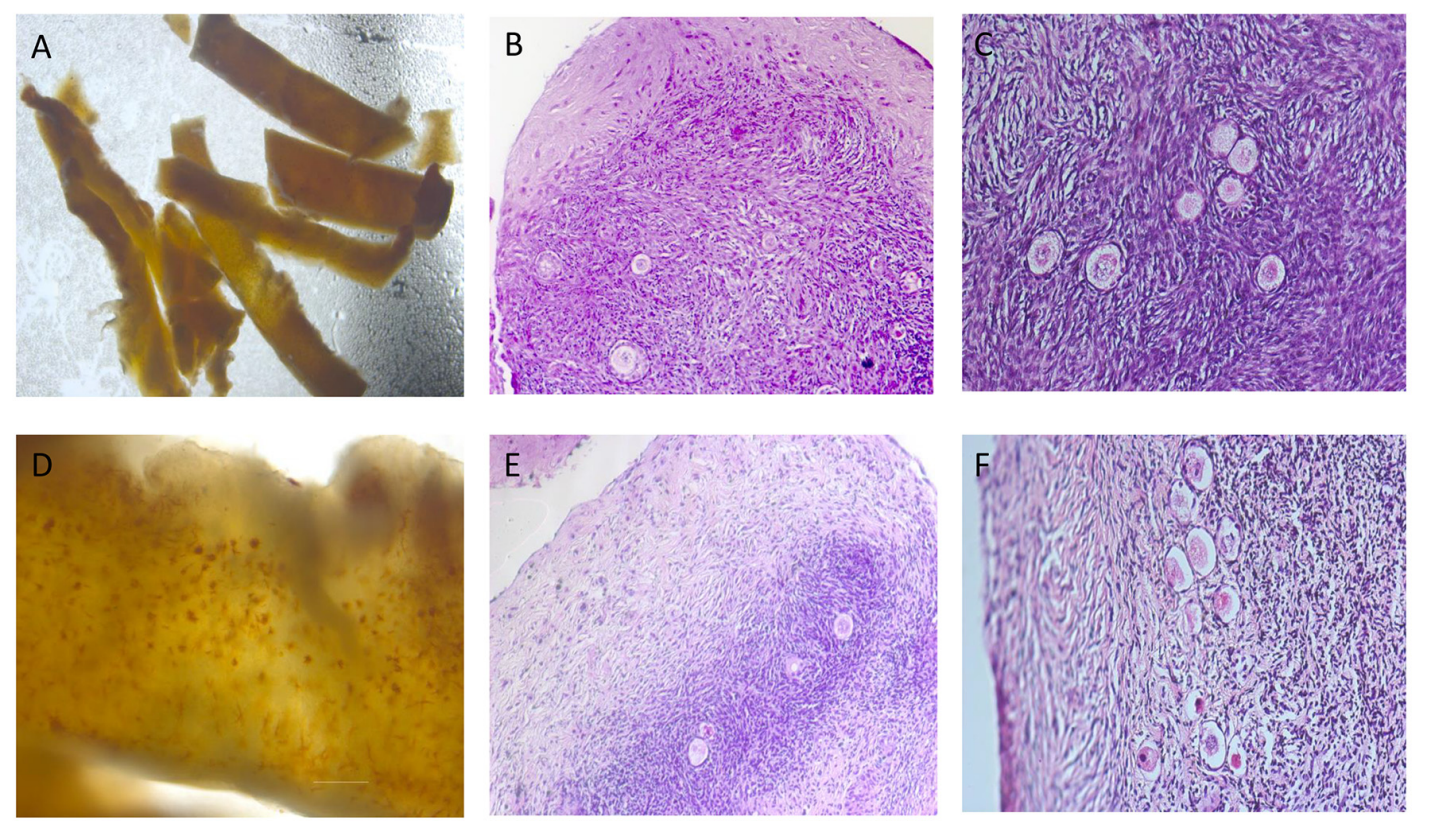
Morphology of ovarian tissue. **(A)** Neutral red-stained ovarian tissue just after thawing: various levels of immature follicles (10x magnification); **(B)** Hematoxylin-eosin (HE)-stained ovarian tissue after thawing and 7 days of *in vitro* culture: fibrosis at the outer edge of cortical slice (100x magnification); **(C)** HE-stained ovarian tissue after thawing and 7 days of *in vitro* culture: viable follicles (200x magnification); **(D)** Neutral red-stained ovarian tissue just after thawing: various levels of immature follicles (40x magnification); **(E)** HE-stained ovarian tissue after thawing and 7 days of *in vitro* culture: fibrosis at the outer edge of cortical slice (100x magnification); **(F)** HE-stained ovarian tissue after thawing and 7 days of *in vitro* culture: necrotic follicles (200x magnification). Note: only part of tissue (2A) was used for experiments described here.

Morphology of fresh fragments of ovarian tissue (**Figure 2A**) after seven days of *in vitro* culture, was changed (**Figures 2B-F**). It appears in a dense fibrotic capsule as well as in slightly decreased volume. The originally sharp edges of the ovarian cortical pieces have become rounded and are no longer angular.

**Figures 2B, E** demonstrate different degrees of fibrosis occurring in ovarian cortical slices after *in vitro* culture. Cortical slices have a higher degree of fibrosis, more follicular necrosis, compressed living space of the follicles and affecting development of mature follicles (**Figure 2E**). It was noted activated primordial follicles and cubic primary follicles with granulosa cells entering the growth and development trajectory, as well as some secondary follicles with multiple layers of granulosa cells and follicular membrane cells (**Figure 2C**). It was also detected necrotic degeneration of primordial follicles and the morphology and distribution of interstitial cells within fibroses tissue masses, characterized by the disordered arrangement of interstitial cells, necrosis of follicles, and flattened granulosa cells (**Figure 2F**).

**Figure 3:**
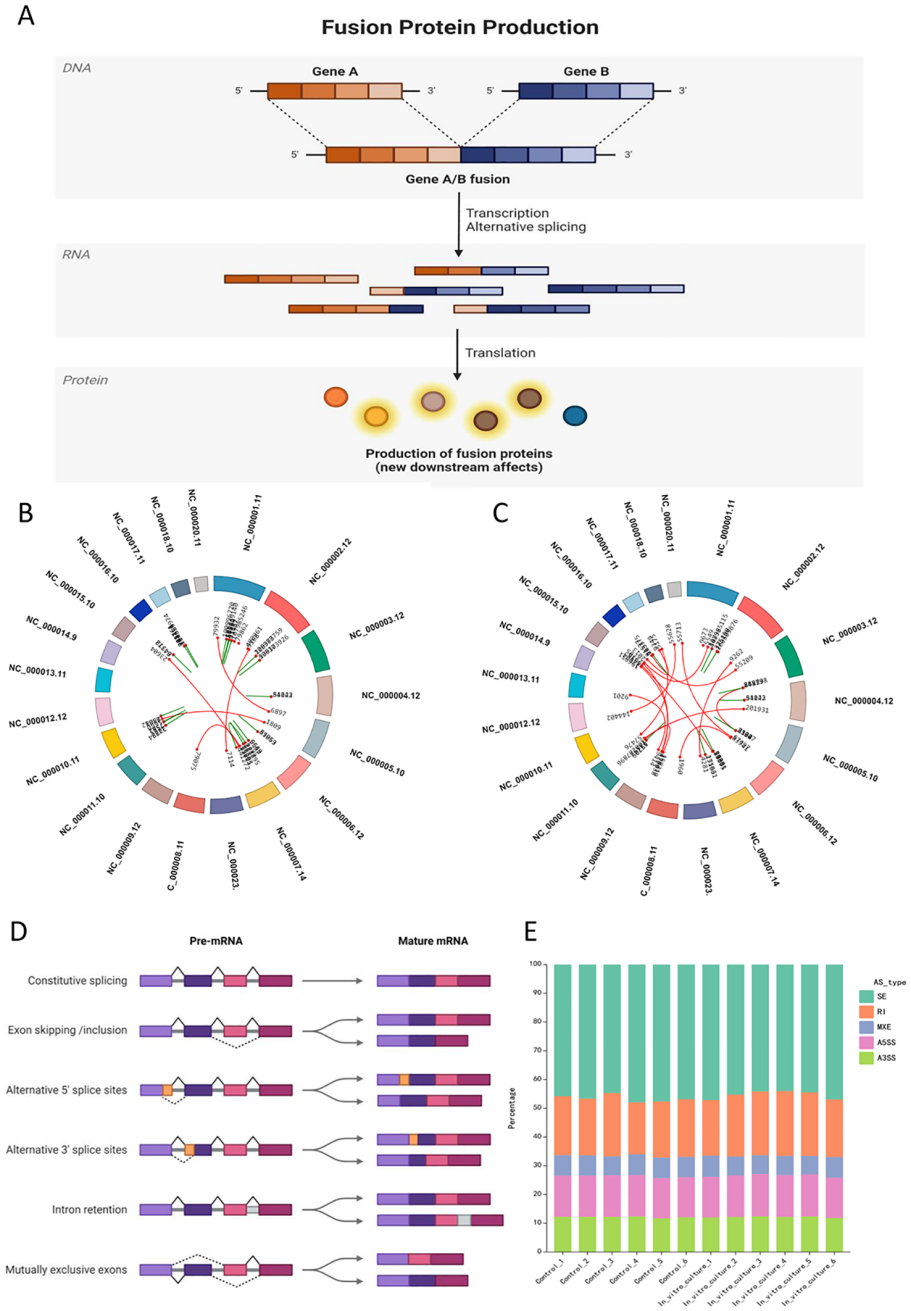
Specific events at the DNA and RNA levels in ovarian tissue after *in vitro* culture: gene fusion and alternative splicing. **(A)** Schematic illustration of gene fusion at the DNA level, where gene fusion events affect the expression from DNA to downstream proteins; **(B, C)** Circos plots showing examples of gene fusion events in post-culture samples compared to the control group; **(B)** Gene fusion events in In_vitro_culture_1; **(C)** Gene fusion events in In_vitro_culture_2; **(D)** Classification of alternative splicing events and schematic illustration of sequence alteration sites at the mRNA level; **(E)** Stacked bar chart showing the proportion of alternative splicing events in each sample group, with the X-axis representing the sample names and the Y-axis representing the percentage of different types of alternative splicing in the respective samples, with each color representing a type of alternative splicing.

It was detected the variable splicing classification and proportion for all samples, with each group of samples showing broadly similar data (**Figures 3A-E**). However, variable splicing occurred in all groups, and there was no significant difference in the types and proportions of variable splicing between two groups. After *in vitro* culture, the frequency of SE (skipped exon) decreased, while the frequency of RI (retained intron) increased (**Figures 3A-E**).

Differences in types and proportions of variable splicing may affect disease progression and prognosis. Different splicing events may lead to various gene expression. Exon skipping in genes such as TUBB6, FGFR1, cAMP-dependent protein kinase inhibitor gamma (PKIG), and METTL5 was observed, which could lead to the development of cancer or affect a normal function of nervous system (**Figures 3A-E**).

**Figure 4:**
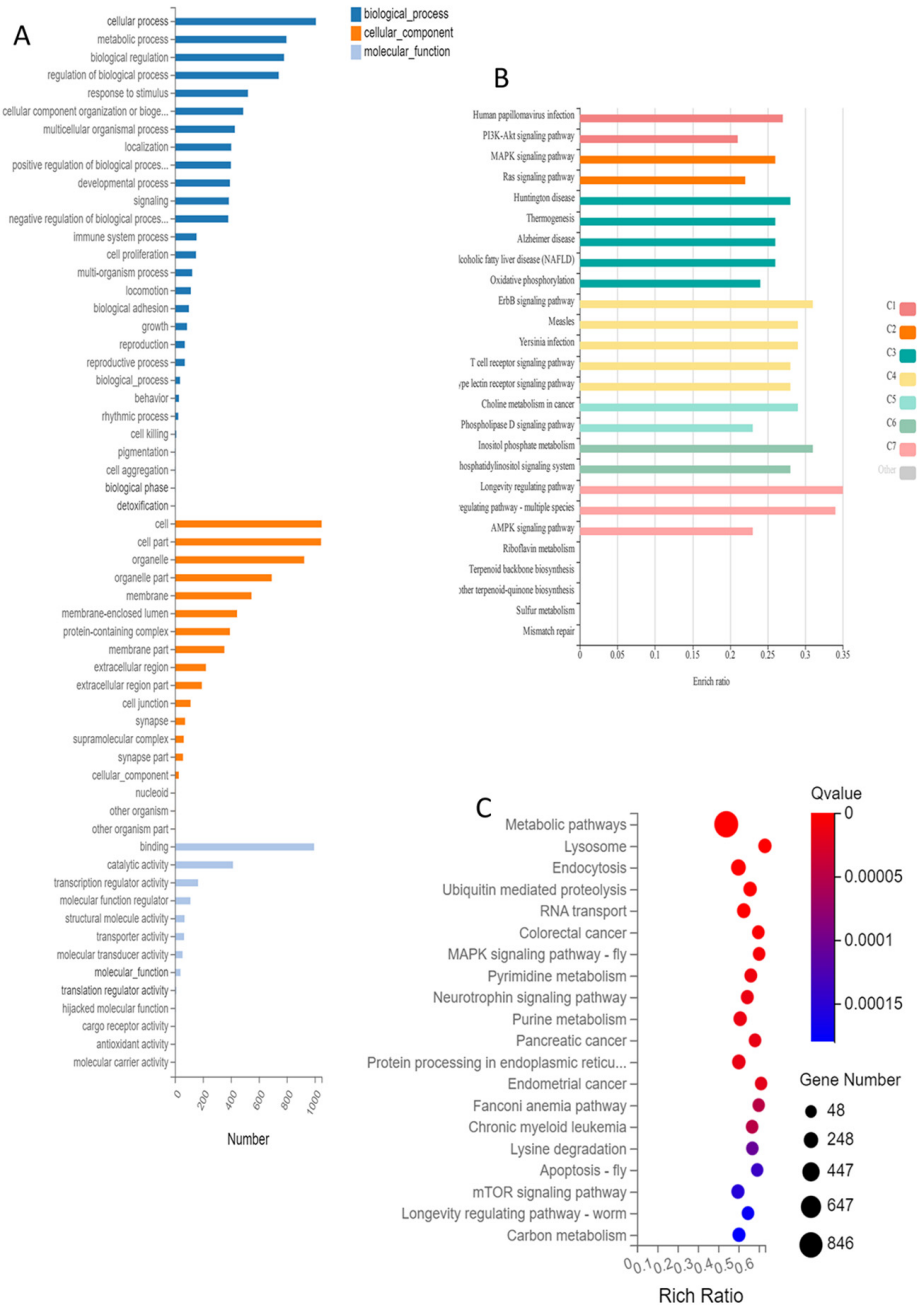
Functional clustering and database annotation of genes undergoing alternative splicing events and SNP events in ovarian tissue after *in vitro* culture. **(A)** Visualization of GO enrichment annotations for differential alternative splicing events; **(B)** KEGG clustering and KOBAS classification of functional SNP/InDel mutation sites: C1-C7; **(C)** Visual bubble chart of KEGG clustering analysis results for differential AS. (C1) HPV-PI3K-signaling; (C2) MAPK-Ras signaling; (C3) neurodegenerative diseases-oxidative phosphorylation; (C4) signaling pathway of receptors on infectious and immune cells; (C5) choline metabolism-phospholipase D; (C6) inositol phosphate; (C7) regulating pathway.

Alternative Splicing (AS) analysis in the Gene Ontology (GO) covers cellular and metabolic processes, biological regulation, and response to stimulus. In the Cellular Component category, it includes cell, cell part, organelle, organelle part, and membrane. In the Molecular Function category, it mainly covers: binding, catalytic activity, transcription regulator activity, molecular function regulation, and structural molecular activity (**Figure 4A**).

Functional Single Nucleotide Polymorphism (SNP)/Insertion-Deletion (InDel) mutation sites are primarily enriched in Human Papillomavirus (HPV) infection, Phosphoinositide 3-kinase (PI3K)-Akt signaling pathway, Mitogen-Activated Protein Kinase (MAPK) signaling pathway, Ras signaling pathway, Huntington disease, thermogenesis (**Figure 4B**).

Alternative Splicing is mainly enriched in the following Kyoto Encyclopedia of Genes and Genomes (KEGG) terms: metabolic pathways, lysosomes, endocytosis, ubiquitin mediated proteolysis as well as RNA transport (**Figure 4C**).

**Figure 5:**
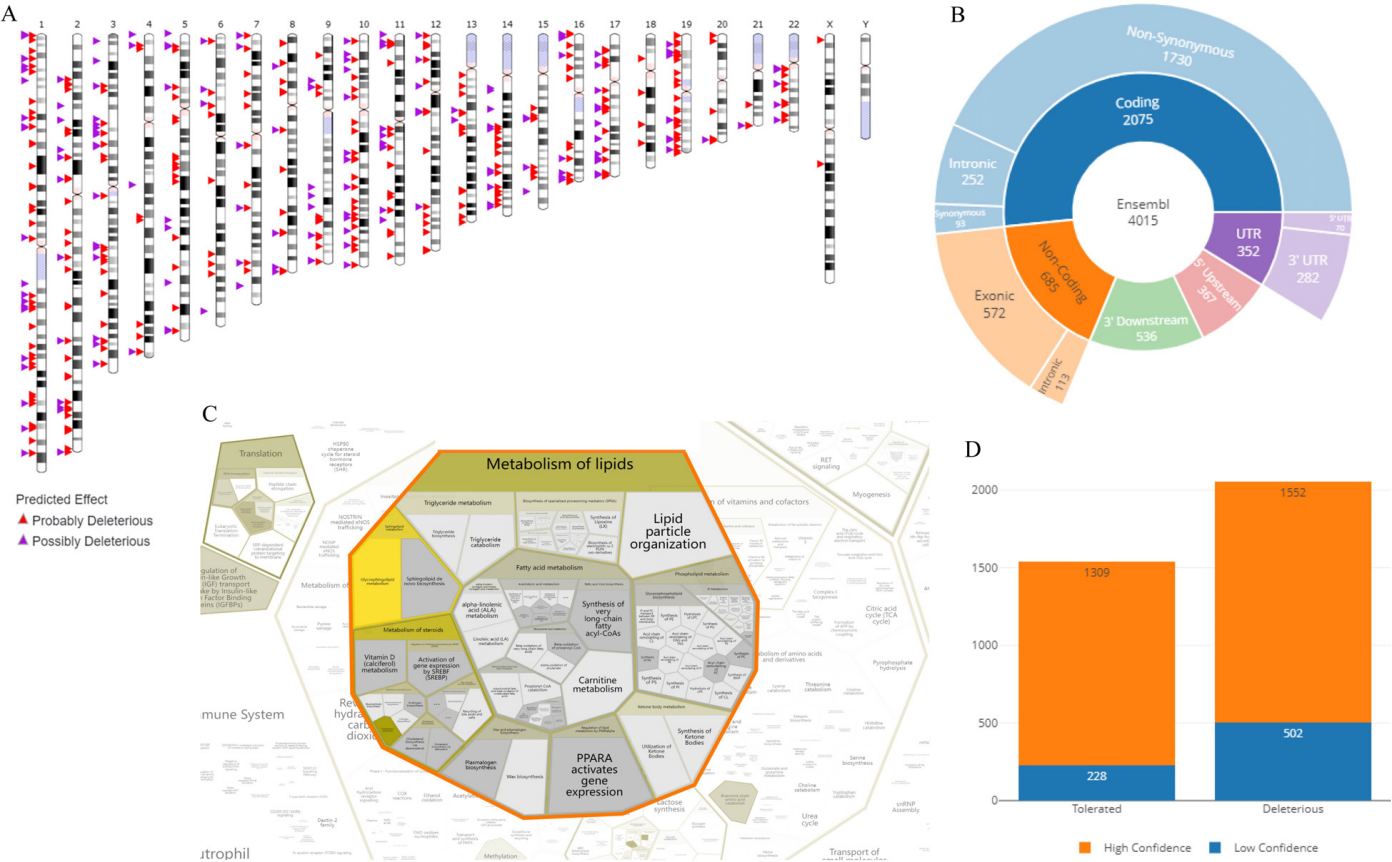
Annotation analysis of suspected deleterious SNP/InDel events. **(A)** Visualization of specific chromosomal locations and distribution of suspected deleterious SNP/InDel events; **(B)** Pie chart visualization of the distribution of SNP/InDel occurrence sites on DNA; **(C)** Bubble chart from the Reactome database for the top term of enriched clustering annotations of SNP/InDel: lipid metabolism, lipid particle organization, fatty acid metabolism, and formation of long-chain fatty acids; **(D)** Stacked bar chart shown the classification and data comparison of suspected deleterious mutation sites of SNP/InDel.

It is shown that the suspected deleterious events of SNVs are distributed on each chromosome after *in vitro* culture. Among them, there are 2075 mutations in the coding sequence and 572 suspected deleterious events in the exon region (**Figure 5**). They are mainly distributed in the lipid metabolism pathway, including the activation and expression of the PPARA gene, and lipid particle organization. It is also detected that there are 1552 high-confidence deleterious sites and 502 low-confidence deleterious sites in the number of gene mutations after *in vitro* culture; there are 1309 high-confidence tolerated sites and 228 low-confidence tolerated sites (**Figure 5**).

## 4 Discussion

Fragments just after operation (“fresh”) were not presented in experiments because for transplantation were used only cryopreserved (frozen and thawed) ovarian tissue. In that way the effectiveness of *in vitro* culture of “fresh” cells is not actual. We need to do *in vitro* culture of cells after cryopreservation to answer two questions:

1. What is the quality of the ovarian tissue in this particular patient in general?
2. What is the quality of ovarian tissue in this particular patient after cryopreservation? How good is the whole cryopreservation process?

We can answer the first question without *in vitro* culture, when a piece of ovarian tissue is fixed and assessed immediately after surgery. In this case, we can see the morphology of this tissue, primarily the morphology of the follicles. But the fact is that for future transplantation we only have cryopreserved tissue that will be transplanted immediately after thawing, without *in vitro* culturing for one week.

We can confidently state that the optimal development of transplanted ovarian tissue will be observed in the patient’s “native” organism, i.e. after thawing and transplantation of such tissue. In fact, more optimal conditions for the further development of transplanted cells than in organism simply cannot exist. The aim of *in vitro* culture technology is to ensure that this technology maximally “copies” the parameters of the external environment after transplantation of these cells to the organism of patient.

We have used the only partial technological separation of medulla from cortex during preparation of ovarian fragments for cryopreservation. It is because the following processes occur after thawing and re-transplantation of ovarian tissue: 1) the cortex with follicles does not have blood vessels and the nutrition of these follicles occurs according to a diffuse principle; 2) for the integration of transplanted tissue, vessels are needed for formation of anastomoses; 3) blood vessels are contained in the medulla in the form of small vessels and stem cells partially differentiated into vessels; 4) complete removal of the medulla is a direct path to necrosis of the transplanted tissue.

For cryopreservation we used relatively large fragments of tissue. It is because cryobiology indicates that for slow (conventional, programmable) freezing, the shape and size of tissue fragments are not only insignificant, but do not play any role at all. Any fragment of ovarian tissue up to 4 mm thick (we use to 1.5 mm) will be completely saturated with permeable cryoprotectants (especially DMSO) within 5 min. from the beginning of equilibration, and this will happen regardless of the area of this fragment.

In our experiments, for thawing we have used 100°C water bath. For demonstration of this technological parameter the following descriptions can be used: 1) when thawing in boiling water, it is used a tampon that isolates the tissue fragment from the bottom of cryovial, which guarantees no overheating (**Figure 1**); 2) the temperature of the tissue fragment after thawing does not rise above +14°C. For illustration: the cryo-vial after being removed from the boiling water after 1 sec. feels cold. This technique is used because a tenet of classical cryobiology states that all types of cells and tissues, regardless of cryopreservation method, must be thawed as quickly as possible.

In fact, fibroblasts, which are in the cortical layer of fragments, have more growth energy than follicular cells.

When a tissue fragment is attached to the bottom of a Petri dish, fibroblasts form a monolayer and their growth energy increases. This in turn leads to the suppression of other cells. Frequent replacement of liquid in a Petri dish is accompanied by frequent movement of the fragment along the bottom of the Petri dish and fibroblasts do not form a monolayer.

Upon microscopic examination of HE-stained sections, it was observed that the encapsulation of tissues cultured *in vitro* is dense, featuring a fibrous capsule that could influence the growth and development of follicles at the cortical cutting edges. Nonetheless, this phenomenon does not seem to hinder follicular development in the central inner parts. This prompts the question: which scenario yields better growth, tissues with dense fibrous encapsulation or those without encapsulation, especially at the edges of a gel scaffold? To address this inquiry, further tests and investigations are warranted in the future.

Recent studies have elucidated significant genetic and epigenetic alterations in gametes and embryos during *in vitro* culture, which hold importance for human assisted reproduction. El Hajj and Haaf highlighted profound epigenetic changes (25), while Kuijk et al. reported increased mutation rates in *in vitro* cultured stem cells, including pluripotent and adult types, primarily attributed to oxidative stress (26). These changes often result in genomic alterations in cells, raising concerns regarding their application in regenerative medicine. Consequently, further research is imperative to comprehend and mitigate these changes, with a specific focus on mutation sites and epigenetic modifications during embryonic development.

Alternative splicing, a process occurring during gene transcription, enables a single gene to undergo splicing, generating multiple distinct mRNA variants, each encoding a unique protein (27). Traditionally, splice isoforms have been categorized based on exon quantity and sequential arrangement (28). However, contemporary research has shifted toward alternative methodologies for splice isoform classification, incorporating criteria such as the nature of splicing events, functional dynamics of splicing factors, and metrics related to ribosomal stalling (29). Beyond splicing factors, the spliceosomal machinery is intricately regulated by an array of transcriptional regulatory elements, non-coding RNAs, and a diverse spectrum of molecular entities (30).

The pathogenesis of numerous diseases has been associated with the production of specific splice isoforms, with aberrant expression profiles of certain splicing factors implicated in various pathologies (31). For instance, SF3B1, a crucial constituent in the RNA splicing cascade, when mutated, disrupts normal splicing mechanisms, resulting in aberrant mRNA and protein synthesis, a phenomenon commonly observed in malignancies such as chronic lymphocytic leukemia (CLL) and myelodysplastic syndromes (MDS) (32).

In the realm of therapeutics, small molecule agents like Pladienolide B and its analogs, which target SF3B1 and analogous splicing factors, are being rigorously investigated for their potential efficacy in treating malignancies characterized by SF3B1 mutations (33, 34). Additionally, in the context of non-small cell lung cancer (NSCLC), the use of small molecule inhibitors like H3B-8800, targeting splicing events associated with mutations in splicing factors such as SRSF2, SF3B1, and U2AF1, represents a novel strategy to disrupt the splicing apparatus and attenuate the proliferation of neoplastic cells (35, 36).

Gene fusion refers to the merging of two or more genes under certain conditions, resulting in a new protein-coding sequence. The mechanisms of gene fusion can be categorized into chromosomal structural variations and transcription/splicing abnormalities, primarily including three types: translocation, involving the transfer of chromosome fragments between chromosomes. Insertion, wherein a chromosome fragment is inserted into a new gap on the same or another chromosome and Inversion, characterized by the 180-degree rotation of a chromosome fragment. For example, EML4-ALK is generated by inversion, serving as one of the driver genes in non-small cell lung cancer (37, 38).

In this study, gene fusion events notably increased after *in vitro* culture, particularly intrachromosomal fusions. This augmentation could be attributed to chromosomal structural changes induced by cellular stress and mechanical instability resulting from fibrosis. However, due to the limited nature of this stress, it did not lead to a higher occurrence of gene fusion events between different chromosomes. Transcriptomic fusion can significantly impact gene expression levels and cellular function. Additionally, related studies have indicated that the rise in gene fusion events in ovarian tissue due to *in vitro* culture also occurs in gamete and zygote *in vitro* cultures (39).

Various *in vitro* culture techniques in assisted reproductive technology, including but not limited to IVM and blastocyst culture, extend the duration of human reproductive cell growth and development outside the body, thereby increasing the frequency of gene fusion events and subsequently elevating the lifetime cancer risk for post-birth offspring (40). Transcript fusion may alter the expression levels of the fused genes and/or the structure and function of the encoded proteins, thereby affecting cell functions. A deeper understanding of the regulatory mechanisms of fused genes can unveil more profound insights into gene expression and regulation. Transcript fusion is a significant outcome in transcriptome sequencing, crucial for enhancing our understanding of gene regulatory mechanisms, diagnosing and treating diseases, and refining transcriptome annotations (41).

InDels (Insertions-Deletions) and SNPs (Single Nucleotide Polymorphisms) are common forms of genetic variation. An InDel refers to an insertion or deletion of one or more bases in the genetic sequence, while a SNP involves the substitution of a single base with another. InDels are more likely to occur than SNPs due to the absence of a point mutation requirement, which is necessary for SNPs. Moreover, InDels generally have a more pronounced impact than SNPs as they can cause relatively larger frame shifts, altering the gene’s reading frame and subsequently changing the protein sequence and structure. SNPs are more suitable for broad distribution analysis across the genome and population genetic studies due to their involvement in single nucleotide variations. InDel and SNP variations are often investigated in tissues with a higher genetic predisposition to diseases such as cancer, neurological disorders, and cardiovascular diseases (42, 43). In the results, the distribution of InDels and SNPs in the *in vitro* cultured group significantly differed from that in the control group, exhibiting a higher frequency of occurrence. This suggests an increased likelihood of congenital defects (44, 45).

This study acknowledges inherent limitations stemming from the scarcity of human gamete samples (46). Given their invaluable nature, the limited quantity of available samples for research remains a persistent bottleneck in this field. Additionally, the academic composition of the research team presented a missed opportunity in integrating extensive bioinformatics, impeding the potential development of comprehensive machine learning algorithms and the establishment of relevant databases (47). However, with the anticipated convergence of multidisciplinary fields and the evolution of interdisciplinary sciences, these opportunities are expected to materialize in the future.

## 5 Conclusion

In conclusion, long-time *in vitro* culture of human ovarian tissue results in substantial changes in its morphology and genetic alteration.

## Data Availability

The original contributions presented in the study are publicly available. This data can be found here: https://www.ncbi.nlm.nih.gov/bioproject/PRJNA932020#.
